# Enhancing sustainability in medical product supply chains: The role of remanufacturing and government subsidies

**DOI:** 10.1016/j.heliyon.2024.e33963

**Published:** 2024-07-04

**Authors:** Yang Bai, Yanjing Liu, Shichao Han, Wenqi Song

**Affiliations:** a Business School, Shandong University of Technology, Zibo, China; b Department of Ophthalmology, Zibo Central Hospital, Zibo, China

**Keywords:** Medical supply chain, Pandemic response, Remanufacturing, Government subsidies, Sustainability

## Abstract

This study explores the impact of government subsidies on the production dynamics within the medical product supply chain, particularly focusing on the remanufacturing of medical goods. Amidst the backdrop of the COVID-19 pandemic, which has underscored the critical shortages in medical supplies, our research delves into the adoption of remanufacturing practices by medical product manufacturers as a strategic response to these shortages and environmental concerns. We investigate how government subsidies influence the production volumes of original manufacturers and remanufacturers and examine the competitive interplay between newly manufactured and remanufactured medical products. Through the development of three production game models—Scenario B (manufacturers produce both new and refurbished products), Scenario N (separate production of new and refurbished products by manufacturers and remanufacturers, respectively), and Scenario C (similar to Scenario N but includes a certification fee paid by remanufacturers to original manufacturers)—we analyze the strategies that could mitigate supply deficiencies during medical crises. Our findings indicate that the certification strategy (Scenario C) not only yields the highest total production of medical products but also offers a viable solution to enhance the sustainability of the entire medical production system by alleviating supply chain disruptions. Furthermore, we discuss the managerial implications of our results, emphasizing the potential of a joint remanufacturing strategy to stabilize the supply chain and foster environmental conservation. Lastly, we highlight our study's limitations and suggest future research directions, particularly concerning the variability in product quality and the reliance on government subsidies. This research contributes to a nuanced understanding of green remanufacturing within the pharmaceutical supply chain, offering insights for manufacturers, remanufacturers, and policymakers aiming for sustainable industry practices.

## 1 Introduction

The COVID-19 pandemic has profoundly affected production and daily life across various sectors worldwide. Consequently, devising effective responses to medical emergencies has become a paramount challenge for governments and global enterprises. A critical component in combating such outbreaks is the assurance of sufficient medical supplies. However, the medical supply stockpile in many cities globally is less than optimal, with notable disparities evident in Chinese cities where only 40 % maintain adequate medical safety stocks [1]. These deficiencies have economic ramifications. For instance, China incurs substantial economic losses annually due to expired medical supplies, representing financial wastage and resource inefficiency. This predicament is not unique to China. A 2014 U.S. Department of Homeland Security (DHS) inspection revealed that 81 % of antiviral drugs are nearing expiration [2].

Similarly, New Zealand had to dispose of approximately 1.5 million doses of outdated anti-flu medication from its reserves, amounting to a retail value loss of $110 million [3]. Australia confronts its challenges, discarding medical supplies worth $200 million past their effective life into landfills annually [4]. To enhance the robustness of the pharmaceutical supply chain and minimize resource wastage, several medical product manufacturers are increasingly adopting the practice of remanufacturing medical products [5]. G.E. Healthcare, a pioneer in this domain, has operated a dedicated refurbishment unit for two decades, branded as GoldSeal [6]. Similarly, Philips established its own medical equipment refurbishment venture, the Diamond Select project [7]. Siemens Healthcare contributes to this trend with its ecoline initiative, focusing on equipment refurbishment [8].

Furthermore, Canon Healthcare has entered this arena with its second life business, which specializes in refurbished medical equipment [9]. Companies like G.E. Healthcare and Siemens Healthineers refurbish medical imaging equipment such as MRI machines and C.T. scanners, ensuring they perform as new [5,7]. Similarly, FDA-regulated reprocessors like Stryker's Sustainability Solutions and Medline ReNewal reprocess single-use devices, including orthopedic saw blades and endoscopic trocars, by cleaning, disinfecting, and repackaging them, significantly reducing waste and costs [10,11]. Additionally, firms such as Philips and Zoll Medical remanufacture life-saving devices like defibrillators and surgical instruments, often sent for refurbishment to extend their lifespan and functionality [7,12]. These practices support environmental sustainability by minimizing waste and raw material use and help make healthcare more accessible and affordable. This shift towards remanufacturing signifies a crucial step in promoting sustainability and efficiency within the healthcare sector.

Meanwhile, as public awareness of environmental issues and social responsibility grows, various sectors increasingly commit to sustainable management and practices. This commitment is particularly evident in the context of pharmaceutical residues, which have been recognized as environmental pollutants. Understanding the implications of using expired or unsuitable medications is critical. Such awareness contributes to the efficient production and reprocessing of excess medicines and ensures their steady availability during medical emergencies while reducing pharmaceutical waste's environmental impact. Consequently, many governments are providing financial support to the pharmaceutical industry. This aid facilitates the recycling and remanufacturing of medicines, ensuring both the sustainability of these practices and the continued availability of essential medications.

To secure government subsidies, medical product manufacturers and remanufacturers increasingly produce and remanufacture medical goods. This trend is particularly evident among remanufacturers, who have started recycling and remanufacturing expired medical products. In a strategic move to garner additional subsidies, some large supply companies are exploiting their recycling capabilities to remanufacture and rebrand these products, thereby challenging the dominance of original manufacturers in the downstream market. The entry of remanufacturers into the medical product remanufacturing sector poses a significant threat to the monopolistic position traditionally held by original manufacturers. Nonetheless, the established brand strength of these original manufacturers often results in a consumer preference for their products. This preference is reinforced by the perception that certified refurbished medical products from original manufacturers are more reliable and of higher quality. A contentious issue in this context is the acceptance of quality certification from original manufacturers. The decision to endorse or provide such certification remains debatable for consumers and brand owners. This uncertainty reflects the complex dynamics between brand reputation, product quality, and market competition in the medical products industry.

With these subsidies determined by the quantity of remanufactured components, manufacturers enhance their marginal revenue through remanufacturing and influence the production volume of new items. Concurrently, this uptick in remanufactured product output inevitably impacts the market for new medical products.

In light of the context mentioned above, this study addresses the subsequent research inquiries.

1. How do government subsidies influence the production volume of original medical product manufacturers and remanufacturers?
2. In a competitive market where traditional products vie against remanufactured medical items, does certification as a remanufacturer yield mutual benefits for medical product manufacturers?
3. Which situation is most favorable in mitigating medical supply deficiencies during medical crises?

**Fig.1:**
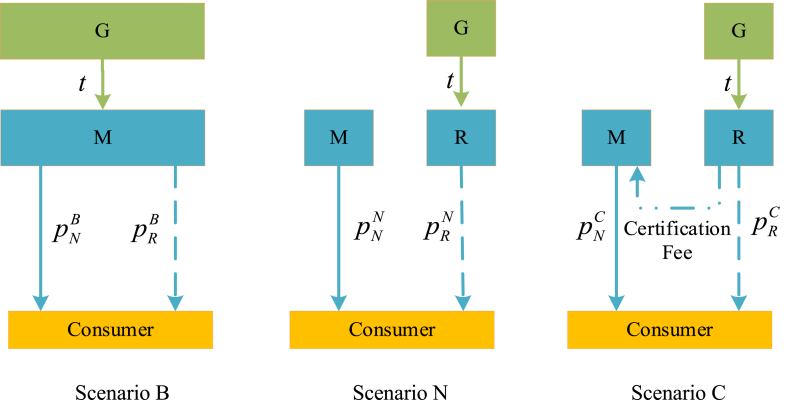
Medical product channel structure. Scenario B involves joint production of new and refurbished products, Scenario N involves independent production by manufacturers and remanufacturers, and Scenario C includes independent production and remanufacturer certification fees.

To solve these problems, we devised three production game models to address medical product shortages: Scenario B, where manufacturers produce both new and refurbished products; Scenario N, where manufacturers produce new products and remanufacturers handle refurbished ones; and Scenario C, akin to Scenario N but with remanufacturers paying a certification fee to original manufacturers to minimize brand differentiation (see Fig. 1).

Prior research in green remanufacturing has predominantly concentrated on firms' production-related decisions, encompassing aspects like retail pricing, investment in eco-friendly initiatives, and the output of green products, with scant attention to the realm of sustainable pharmaceutical supply chains. This study delves into the remanufacturing of medical products, specifically examining the ramifications of government subsidies on this process. Additionally, it investigates the effects of remanufacturer market encroachment and the adjustments in certification decisions by original medical product manufacturers on the strategic decisions regarding supply chain channel structures. Our findings reveal several intriguing points: (1) while government subsidies for remanufactured products do boost the production of these items, their influence on the output of newly manufactured medical products in a given scenario (referred to as Scenario B) might be marginal; (2) the strategy C (certification) results in the highest total production output for medical products; (3) under the Scenario C (certification) strategy, manufacturers are capable of compensating for potential losses in the absence of government subsidies, by leveraging certification fees paid by the remanufacturers. These insights contribute to a nuanced understanding of the interplay between government policies, certification strategies, and production dynamics in the context of green remanufacturing within the pharmaceutical supply chain.

The structure of this paper is organized as follows: Section 2 presents a comprehensive literature review. Section 3 delineates the proposed model and elaborates on the optimal policy formulations. Section 4 provides the concluding remarks. Proofs for all claims and propositions can be found in the Appendix.

## 2 Literature review

This paper scrutinizes the strategies pertinent to the sustainable supply chain and remanufacturing of medical products. Commencing with a brief literature review on the sustainability of medical supply chains, the discourse further navigates through an analytical perusal of government subsidies and the methodology of selecting channel structures.

Kargar et al. [13] proposed a model prioritizing sustainability and cost-efficiency in medical waste management. Weraikat et al. [14] recommended a decentralized negotiation process to enhance the collection and recycling of unused medicines within the pharmaceutical industry. Confronting the spike in demand for dietary supplements during pandemics, Datta et al. [15] presented a mathematical model to alleviate manufacturing constraints. They proposed the promising use of blockchain technology as a resolution. Zhang et al. [1] underscored the requirement for substantial medical supplies during epidemics, emphasizing the significance of financial reserves in tandem with physical stockpiles. They posited that capital reserves may offer a more optimal choice in certain instances of demand uncertainty.

Furthermore, Tat and Heydari [16] advanced a collaborative model that included a donation program and take-back strategy to curtail medicine waste in the pharmaceutical supply chain. Their model was proven to augment both profits and sustainability while diminishing waste. Levner and Herbon [17] devised a mathematical model to minimize the costs of vaccine storage and distribution, considering environmental considerations. Their findings suggest that investment in environmentally-friendly practices can be cost-effective and optimize vaccination plans. While the studies mentioned above encompass a range of topics, from medical waste management and recycling of unused medications to addressing surges in supplement demand and optimizing medical product distribution, they overlook the crucial role of government subsidies. By contrast, our study centralizes its focus on the influence of government subsidies on the sustainability of medical product supply chains.

Our research aligns with the literature on the impact of government subsidies. Huang et al. [18] champion a government-initiated incentive scheme, encouraging the public to return expired drugs to pharmacies for proper recycling. Hua et al. [19] accentuate the necessity for a robust household medication collection system facilitated through government incentives and promotional activities. Tat et al. [20] draw attention to the financial losses from surplus medications, proposing their resale before expiration. To streamline this process, they introduced a contract to coordinate supply chains. Liu et al. [21] delve deeper into recycling strategies and furnish a comprehensive government subsidy model that harmonizes economic, environmental, and social benefits. Subsequently, Tat et al. [16] propose a medicine donation scheme and a take-back plan for surplus medication to bolster public health and corporate responsibility. Fang et al. [22] illustrate how government regulations can stimulate green innovation, citing increased green patents within highly polluting industries. Suhandi and Chen [23] advocate for a closed-loop supply chain model in the pharmaceutical industry, encouraging recycling, reducing prescription drug costs, and increasing government subsidies. They attest to the potential of drug recycling programs for environmental and societal gains. Cumulatively, these studies emphasize the significance of effectively managing residual and expired medications, suggesting diverse government incentive strategies for their collection and recycling. Compared to the literature mentioned above, the distinctive aspect of our research is our discussion of remanufacturers' profits in producing remanufactured medical products across varied channels. This discussion offers a theoretical basis for remanufacturers to choose production channels and enhance profits.

Lastly, our study correlates with the literature on selecting remanufacturing channels. Liu et al. [24] scrutinized how outsourcing strategies influence the sales format choices of private-label brands on e-commerce platforms. Xie et al. [25] discovered that a high external benefit or brand premium inclines manufacturers towards reshoring. If outsourcing is chosen, they tend towards original brand manufacturers with a high brand premium or non-competing contract manufacturers with a low brand premium. Wu et al. [26] concentrated on the sourcing strategies of technology platform developers, considering consumer preferences for products and operating systems (O·S.). Niu et al. [27] delved into the advantages of remanufacturers of remanufactured components establishing their brands, questioning whether Original Equipment Manufacturers (OEMs) should continue sourcing from them or divert to new-component remanufacturers. Wei and Xu [28] suggested that strategies depend on platform fees, consumer preferences, and product quality. Li et al. [29] explored strategies of apparel manufacturers, such as outsourcing or setting up in-house factories, asserting that factors like investment cost and consumer preferences sway these decisions. Hou et al. [30] probed into the determinants that drive OEMs' decisions to shift production to lower-cost regions. They concluded that brand value, competition with remanufacturers, and innovation effectiveness were integral considerations in such decisions. The distinguishing feature of our work compared to the literature mentioned above is our focus on aligning incentives through quality certification between the new brand manufacturer and the remanufacturer. Table 1 presents the contributions and gaps of the research.

**Table 1:** Comparison of this study with related studies.

Literature	Government subsidies	Quality certification	PSC	Periods	Coordination mechanism	Brand premium
Xin et al. [31]				1	✓	
Bai and Song [32]				2	✓	
Peng et al. [33]				1	✓	
Li et al. [34]				2	✓	
Xie et al. [25]	✓			1	✓	✓
Niu et al. [27]	✓			1	✓	✓
Chen et al. [35]	✓		✓	1		
Niu et al. [36]	✓			1	✓	
Niu and Xie [37]		✓		1	✓	
This study	✓	✓	✓	2	✓	✓

Note: PSC: Per-unit sustainability production cost.

## 3 Model

This study proposes three production game models: Scenario B, where manufacturers produce both new and refurbished products; Scenario N, wherein manufacturers produce only new products and remanufacturers oversee refurbished ones; and Scenario C, similar to Scenario N but with remanufacturers paying a certification fee to original producers to minimize brand differentiation. The three different scenarios are shown in Fig. 1.

Assumption 1 In this study, $c_{N}$ and $c_{R}$ represent the manufacturing and remanufacturing costs, respectively. In addition, we consider the marginal production cost of new medical products to be higher than the marginal production cost of remanufacturing, i.e., ${0<c}_{R}<c_{N}\text{.}$ Otherwise, the remanufacturer will be expelled from the remanufacturing market. $p_{N1}^{j}$ represents the price of the new medical products to consumers in the first period. $p_{N}^{j}$ and $p_{R}^{j}$ represent the new and remanufactured medical products to consumers in the second period, respectively (j=B,N,C). The specific model notation and description are presented in Table 2.

**Table 2:** Notations

Symbol	Definition	Symbol	Definition
$p_{N1}^{j}$	Price of the new medical products in the first period	$c_{N}$	New medical products' marginal production cost
$p_{N}^{j}$	Price of the new medical products in the second period	$c_{R}$	Remanufacturing marginal production cost
$p_{R}^{j}$	Price of the remanufacturing of medical products in the second period	m	Remanufactured product disadvantage
$q_{N1}^{j}$	Selling quantity of the new medical products in the first period	f	Certification fee
$q_{N}^{j}$	Selling quantity of the new medical products in the second period	t	Government subsidy
$q_{R}^{j}$	Selling quantity of the remanufacturing medical products	β	Per-unit sustainability production cost
$\pi_{M}^{j}$	Profits of manufacturers remanufacturing medical products in the second period	$\pi_{R}^{j}$	Profits of remanufacturer

Assumption 2 Assuming the new medical products have a brand advantage over the remanufactured ones. This means the brand disadvantage will attenuate the demand for remanufactured medical products [29,36]. Therefore, in this research, we use parameter m reflects the remanufactured product disadvantage.

Assumption 3 The remanufactured product disadvantage m is not negligible and satisfies $m>m^{*}>2\left(1+\beta \right)c_{N}+t-1-2\beta -c_{R}\text{,}$ which ensures that the remanufacturing production quantity in the second period is strictly positive. In addition, the government subsidy t is not negligible and satisfies $t>t^{*}>\frac{c_{N}+4\left(1+2\beta \right)c_{R}-8\beta -5}{4\left(1+2\beta \right)}\text{,}$ which ensures the certification fee is strictly positive (see Appendix for the details).

### 3.1 Under scenario B (Based model)

The manufacturer produces new medical products with the manufacturer's cost $c_{N}$, and the manufacturer develops the near-expired or life-ended medical productions and opts to remanufacture with the cost $c_{R}$, based on which the manufacturer can get the government subsidy t. The manufacturer sets the new medical products' price $p_{N1}^{B}$ to the consumers in the first period. Meanwhile, the manufacturer sells both the new and remanufactured medical products to consumers in the second period at the price $p_{N}^{B}$ and $p_{R}^{B}$, respectively. According to Chen et al. [35], we assume that sustainability effort also affects the unit production cost, i.e., $c=c_{R}+{\;\beta q}_{R}$, which consistent with the law of diminishing marginal returns. In addition, β>0 represents the sustainability production processes that increase the per-unit production cost. Therefore, the profit functions of the manufacturer under scenario B can be given as follows (Equation (1)):

(1) $$\begin{array}{c} \pi_{M}^{B}=\pi_{N}^{B}+\pi_{R}^{B}=\left(p_{N1}^{B}-c_{N}\right)q_{N1}^{B}+\left(p_{N}^{B}-c_{N}\right)q_{N}^{B}+\left(p_{R}^{B}-c_{R}-\beta q_{R}^{B}+t\right)q_{R}^{B} \end{array}$$

Where $p_{N1}^{B}=1-q_{N1}^{B}{,p}_{N}^{B}=1-q_{N}^{B}-q_{R}^{B}\text{,}$ and $p_{R}^{B}=1-q_{R}^{B}-q_{N}^{B}\text{.}$

According to the backward introduction approach, Lemma 1 provides the equilibrium outcomes of the manufacturer under scenario B.

Lemma 1 Under scenario B, the manufacturer's optimal decisions are $q_{N1}^{B}=\frac{1-c_{N}}{2},q_{N}^{B}=\frac{\beta -t-\left(1+\beta \right)c_{N}+c_{R}}{2\beta }$ and $q_{R}^{B}=\frac{t+c_{N}-c_{R}}{2\beta }\text{,}$ respectively. Correspondingly, the profits of the manufacturer are $\pi_{M}^{B}=\frac{t^{2}+2\beta +c_{N}\left(2t-4\beta +c_{N}+2\beta c_{N}\right)-2\left(t+c_{N}\right)c_{R}+c_{R}^{2}}{4\beta }$.

Corollary 1 Under scenario B:

1) $\frac{\partial q_{N}^{B}}{\partial t}=-\frac{1}{2\beta }<0,\frac{\partial q_{R}^{B}}{\partial t}=\frac{1}{2\beta }>0,\frac{\partial \pi_{M}^{B}}{\partial t}=\frac{2t+2c_{N}-2c_{R}}{4\beta }>0\text{.}$
2) $\frac{\partial q_{N}^{B}}{\partial \beta }=\frac{t+c_{N}-c_{R}}{2\beta^{2}}>0,\frac{\partial q_{R}^{B}}{\partial \beta }=-\frac{t+c_{N}-c_{R}}{2\beta^{2}}<0,\frac{\partial \pi_{M}^{B}}{\partial \beta }=-\frac{{\left(t+c_{N}-c_{R}\right)}^{2}}{4\beta^{2}}<0\text{.}$
3) $\frac{\partial q_{N}^{B}}{\partial c_{N}}=\frac{-1-\beta }{2\beta }<0,\frac{\partial q_{R}^{B}}{\partial c_{N}}=\frac{1}{2\beta }>0,\frac{\partial \pi_{M}^{B}}{\partial c_{N}}=\frac{t-2\beta +c_{N}+2\beta c_{N}-c_{R}}{2\beta }<0\text{.}$
4) $\frac{\partial q_{N}^{B}}{\partial c_{R}}=\frac{1}{2\beta }>0,\frac{\partial q_{R}^{B}}{\partial c_{R}}=-\frac{1}{2\beta }<0,\frac{\partial \pi_{M}^{B}}{\partial c_{R}}=-\frac{t+c_{N}-c_{R}}{2\beta }<0\text{.}$

Corollary 1 presents a sensitivity analysis in the context of scenario B. This assessment is intuitively given that governmental incentives, such as subsidies, encourage the production of more remanufactured medical products. As these subsidies grow, it's foreseeable that manufacturers will scale up production correspondingly. However, a countervailing factor is that the surge in remanufacturing impacts the manufacturer's downstream market negatively, prompting a reduction in production. Despite the dwindling demand for new medical products, the manufacturer can offset these losses with the rising demand for remanufactured medical items. However, an upsurge in the production of remanufactured products results in a corresponding rise in per-unit production costs, leading manufacturers to reduce the volume of remanufactured medical production while increasing the output of new medical items. This shift tends to curtail the manufacturer's profits. Meanwhile, the high new (remanufactured) production cost leads to a large increase in the demand for quantities of remanufactured (new) products. This interplay ultimately diminishes the manufacturer's overall profits.

### 3.2 In Scenario N (No Certification)

The manufacturer produces new medical products at a cost denoted as $c_{N}$, and sells these newly produced medical products to consumers at the price $p_{N1}^{N}$ in the first period. Simultaneously, the remanufacturer processes near-expired or end-of-life medical products, opting for remanufacturing at a cost marked as $c_{R}$. These remanufactured medical products are then sold directly to consumers at a selling price represented by $p_{R}^{N}$. Meanwhile, the manufacturer sells the new medical products to consumers in the second period at the price $p_{N}^{N}$. To promote sustainable development, the government offers a subsidy, t, to the remanufacturer. Consequently, the profit functions for both the manufacturer and the remanufacturer under Scenario N are defined as follows (Equations (2), (3), (4)):

(2) $$\begin{array}{c} \pi_{M1}^{N}=\left(p_{N1}^{N}-c_{N}\right)q_{N1}^{N} \end{array}$$

(3) $$\begin{array}{c} \pi_{M2}^{N}=\left(p_{N}^{N}-c_{N}\right)q_{N}^{N} \end{array}$$

(4) $$\begin{array}{c} \pi_{R}^{N}=\left(p_{R}^{N}-c_{R}-\beta q_{R}^{B}+t\right)q_{R}^{N} \end{array}$$

Where $p_{N1}^{N}=1-q_{N1}^{N}{,p}_{N}^{N}=1-q_{N}^{N}-q_{R}^{N}$ and $p_{R}^{N}=1-q_{R}^{N}-q_{N}^{N}-m\text{.}$ The parameter m reflects the remanufactured product disadvantage. According to the backward introduction approach, the detailed equilibrium decisions of the manufacturer and remanufacturer are presented in Lemma 2.

Lemma 2 Under scenario N, the manufacturer and remanufacturer's optimal decisions are $q_{N1}^{N}=\frac{1-c_{N}}{2}\text{,}$ $q_{N}^{N}=\frac{1+m-t+2\beta -2\left(1+\beta \right)c_{N}+c_{R}}{3+4\beta }\text{,}$ and $q_{R}^{N}=\frac{1-2m+2t+c_{N}-2c_{R}}{3+4\beta }\text{,}$ respectively. Correspondingly, the profits of the manufacturer and remanufacturer are $\pi_{M}^{N}=\frac{1}{4}{\left(1-c_{N}\right)}^{2}+\frac{{\left(1+m-t+2\beta -2\left(1+\beta \right)c_{N}+c_{R}\right)}^{2}}{{\left(3+4\beta \right)}^{2}}$ and $\pi_{R}^{N}=\frac{\left(1+\beta \right){\left(1-2m+2t+c_{N}-2c_{R}\right)}^{2}}{{\left(3+4\beta \right)}^{2}}\text{,}$ respectively.

Corollary 2 Under scenario N:

1) $\frac{\partial q_{N}^{N}}{\partial t}=-\frac{1}{3+4\beta }<0,\frac{\partial q_{R}^{N}}{\partial t}=\frac{2}{3+4\beta }>0,\frac{\partial \pi_{M}^{N}}{\partial t}=-\frac{2\left(1+m-t+2\beta -2\left(1+\beta \right)c_{N}+c_{R}\right)}{{\left(3+4\beta \right)}^{2}}<0,\frac{\partial \pi_{R}^{N}}{\partial t}=\frac{4\left(1+\beta \right)\left(1-2m+2t+c_{N}-2c_{R}\right)}{{\left(3+4\beta \right)}^{2}}>0\text{.}$
2) $\frac{\partial q_{N}^{N}}{\partial \beta }=\frac{2\left(1-2m+2t+c_{N}-2c_{R}\right)}{{\left(3+4\beta \right)}^{2}}>0,\frac{\partial q_{R}^{N}}{\partial \beta }=-\frac{4\left(1-2m+2t+c_{N}-2c_{R}\right)}{{\left(3+4\beta \right)}^{2}}<0,\frac{\partial \pi_{M}^{N}}{\partial \beta }=\frac{4\left(1+m-t+2\beta -2\left(1+\beta \right)c_{N}+c_{R}\right)\left(1-2m+2t+c_{N}-2c_{R}\right)}{{\left(3+4\beta \right)}^{3}}>0,\frac{\partial \pi_{R}^{N}}{\partial \beta }=-\frac{\left(5+4\beta \right){\left(1-2m+2t+c_{N}-2c_{R}\right)}^{2}}{{\left(3+4\beta \right)}^{3}}<0\text{.}$
3) $\frac{\partial q_{N}^{N}}{\partial c_{N}}=-\frac{2\left(1+\beta \right)}{3+4\beta }<0,\frac{\partial q_{R}^{N}}{\partial c_{N}}=\frac{1}{3+4\beta }>0,\frac{\partial \pi_{M}^{N}}{\partial c_{N}}=\frac{-\left(1-c_{N}\right)}{2}-\frac{4\left(1+\beta \right)\left(1+m-t+2\beta -2\left(1+\beta \right)c_{N}+c_{R}\right)}{{\left(3+4\beta \right)}^{2}}<0,\frac{\partial \pi_{R}^{N}}{\partial c_{N}}=\frac{2b\left(1+\beta \right)\left(2-b-2m+2t+bc_{N}-2c_{R}\right)}{{\left(b^{2}-4\left(1+\beta \right)\right)}^{2}}>0\text{.}$
4) $\frac{\partial q_{N}^{N}}{\partial c_{R}}=\frac{1}{3+4\beta }>0,\frac{\partial q_{R}^{N}}{\partial c_{R}}=-\frac{2}{3+4\beta }<0,\frac{\partial \pi_{M}^{N}}{\partial c_{R}}=\frac{2\left(1+m-t+2\beta -2\left(1+\beta \right)c_{N}+c_{R}\right)}{{\left(3+4\beta \right)}^{2}}>0,\frac{\partial \pi_{R}^{N}}{\partial c_{R}}=-\frac{4\left(1+\beta \right)\left(1-2m+2t+c_{N}-2c_{R}\right)}{{\left(3+4\beta \right)}^{2}}<0\text{.}$
5) $\frac{\partial q_{N}^{N}}{\partial m}=\frac{1}{3+4\beta }>0,\frac{\partial q_{R}^{N}}{\partial m}=-\frac{2}{3+4\beta }<0,\frac{\partial \pi_{M}^{N}}{\partial m}=\frac{2\left(1+m-t+2\beta -2\left(1+\beta \right)c_{N}+c_{R}\right)}{{\left(3+4\beta \right)}^{2}}>0,\frac{\partial \pi_{R}^{N}}{\partial m}=-\frac{4\left(1+\beta \right)\left(1-2m+2t+c_{N}-2c_{R}\right)}{{\left(3+4\beta \right)}^{2}}<0\text{.}$

Corollary 2 offers a sensitivity analysis in Scenario N, applying a logic similar to that of Corollary 1. Firstly, as the government increases, the remanufacturer receives a larger subsidy. This benefit incentivizes the remanufacturer to invest more heavily in remanufacturing, leading to fierce competition and a subsequent increase in higher profits. Conversely, this intense competition can diminish the manufacturer's profits. As the volume of remanufactured products grows, per-unit production costs rise, which motivates the remanufacturer to scale down the production of remanufactured medical items. This shift incentivizes the manufacturer to ramp up the production of new medical items, ultimately augmenting the manufacturer's profits while diminishing the remanufacturer's earnings. Secondly, high new (or remanufactured) production costs significantly increase the demand for remanufactured (or new) products. This interplay results in a decrease in overall profits for the manufacturer (or remanufacturer). Lastly, increasing the perceived disadvantages of remanufactured products can diminish remanufacturing incentives, reducing remanufacturers' market share.

### 3.3 Under Scenario C (Certification)

The manufacturer produces new medical products at a production cost denoted as $c_{N}$, and sells these newly produced medical products to consumers at a price signified by $p_{N1}^{C}$ in the first period. In parallel, the remanufacturer repurposes near-expired or end-of-life medical products, opting to remanufacture them at a cost marked as $c_{R}$. These remanufactured medical products are then sold directly to consumers at a selling price denoted as $p_{R}^{C}$. Meanwhile, the manufacturer sells the new medical products to consumers in the second period at the price $p_{N}^{C}$. To promote sustainable development, the government provides a subsidy, t. to the remanufacturer. At the same time, to mitigate the perceived drawbacks of remanufactured medical products, the remanufacturer procures quality certification from the manufacturer and pays a certification fee, f, per unit product. Consequently, the profit functions for both the manufacturer and remanufacturer under Scenario B are defined as follows (Equations (5), (6), (7)):

(5) $$\begin{array}{c} \pi_{M1}^{C}=\left(p_{N}^{C}-c_{N}\right)q_{N1}^{C} \end{array}$$

(6) $$\begin{array}{c} \pi_{M2}^{C}=\left(p_{N}^{C}-c_{N}\right)q_{N}^{C}+fq_{R}^{C} \end{array}$$

(7) $$\begin{array}{c} \pi_{R}^{C}=\left(p_{R}^{C}-c_{R}-\beta q_{R}^{C}+t-f\right)q_{R}^{C} \end{array}$$

Where $p_{N1}^{C}=1-q_{N1}^{C}{,p}_{N}^{C}=1-q_{N}^{C}-q_{R}^{C}$ and $p_{R}^{C}=1-q_{R}^{C}-q_{N}^{C}\text{.}$ According to the backward introduction approach, the detailed equilibrium decisions of the manufacturer and the remanufacturer are presented in Lemma 3.

Lemma 3 Under scenario C, the manufacturer and remanufacturer's optimal decisions are $q_{N1}^{C}=\frac{1-c_{N}}{2}\text{,}$ $q_{N}^{C}=\frac{5-2t+8\beta -\left(7+8\beta \right)c_{N}+2c_{R}}{2\left(5+8\beta \right)}$ and $q_{R}^{C}=\frac{2\left(t+c_{N}-c_{R}\right)}{5+8\beta }\text{,}$ respectively. The certification fee $f=\frac{5+4t+8\left(1+t\right)\beta -c_{N}-4\left(1+2\beta \right)c_{R}}{2\left(5+8\beta \right)}\text{.}$ Correspondingly, the profits of the manufacturer and remanufacturer are $\pi_{M}^{C}=\frac{5+2t^{2}+8\beta +c_{N}\left(2\left(-5+2t-8\beta \right)+\left(7+8\beta \right)c_{N}\right)-4\left(t+c_{N}\right)c_{R}+2c_{R}^{2}}{2\left(5+8\beta \right)}$ and $\pi_{R}^{C}=\frac{4\left(1+\beta \right){\left(t+c_{N}-c_{R}\right)}^{2}}{{\left(5+8\beta \right)}^{2}}\text{,}$ respectively.

Corollary 3 Under scenario C:

1) $\frac{\partial q_{N}^{C}}{\partial t}=-\frac{1}{5+8\beta }<0,\frac{\partial q_{R}^{C}}{\partial t}=\frac{2}{5+8\beta }>0,\frac{\partial f}{\partial t}=\frac{4+8\beta }{2\left(5+8\beta \right)}>0,\frac{\partial \pi_{M}^{C}}{\partial t}=\frac{4t+4c_{N}-4c_{R}}{2\left(5+8\beta \right)}>0,\frac{\partial \pi_{R}^{C}}{\partial t}=\frac{8\left(1+\beta \right)\left(t+c_{N}-c_{R}\right)}{{\left(5+8\beta \right)}^{2}}>0\text{.}$
2) $\frac{\partial q_{N}^{C}}{\partial \beta }=\frac{8\left(t+c_{N}-c_{R}\right)}{{\left(5+8\beta \right)}^{2}}>0,\frac{\partial q_{R}^{C}}{\partial \beta }=-\frac{16\left(t+c_{N}-c_{R}\right)}{{\left(5+8\beta \right)}^{2}}<0,\frac{\partial f}{\partial \beta }=\frac{4\left(t+c_{N}-c_{R}\right)}{{\left(5+8\beta \right)}^{2}}>0,\frac{\partial \pi_{M}^{C}}{\partial \beta }=-\frac{8{\left(t+c_{N}-c_{R}\right)}^{2}}{{\left(5+8\beta \right)}^{2}}<0,\frac{\partial \pi_{R}^{C}}{\partial \beta }=-\frac{4\left(11+8\beta \right){\left(t+c_{N}-c_{R}\right)}^{2}}{{\left(5+8\beta \right)}^{3}}<0\text{.}$
3) $\frac{\partial q_{N}^{C}}{\partial c_{N}}=\frac{-7-8\beta }{2\left(5+8\beta \right)}<0,\frac{\partial q_{R}^{C}}{\partial c_{N}}=\frac{2}{5+8\beta }>0,\frac{\partial f}{\partial c_{N}}=-\frac{1}{2\left(5+8\beta \right)}<0,\frac{\partial \pi_{M}^{C}}{\partial c_{N}}=\frac{2\left(-5+2t-8\beta \right)+2\left(7+8\beta \right)c_{N}-4c_{R}}{2\left(5+8\beta \right)}<0,\frac{\partial \pi_{R}^{C}}{\partial c_{N}}=\frac{8\left(1+\beta \right)\left(t+c_{N}-c_{R}\right)}{{\left(5+8\beta \right)}^{2}}>0\text{.}$
4) $\frac{\partial q_{N}^{C}}{\partial c_{R}}=\frac{1}{5+8\beta }>0,\frac{\partial q_{R}^{C}}{\partial c_{R}}=-\frac{2}{5+8\beta }<0,\frac{\partial f}{\partial c_{R}}=-\frac{2\left(1+2\beta \right)}{5+8\beta }<0,\frac{\partial \pi_{M}^{C}}{\partial c_{R}}=\frac{-4\left(t+c_{N}\right)+4c_{R}}{2\left(5+8\beta \right)}<0,\frac{\partial \pi_{R}^{C}}{\partial c_{R}}=-\frac{8\left(1+\beta \right)\left(t+c_{N}-c_{R}\right)}{{\left(5+8\beta \right)}^{2}}<0\text{.}$

Corollary 3 presents a sensitivity analysis within Scenario C. Firstly, as the government increases the subsidy denoted as t, the remanufacturer receives a larger subsidy. The financial benefit from this subsidy incentivizes the remanufacturer to invest more in sustainable remanufacturing, which increases his order quantity and profits. As the remanufacturer's profits rise, he is likely to pay a higher certification fee to the manufacturer, thus offsetting the manufacturer's losses and creating a win-win situation for both parties. However, a surge in the volume of remanufactured products leads to an increase in per-unit production costs. This prompts the remanufacturer to decrease the quantity of remanufactured medical production, incentivizing the manufacturer to increase the output of new medical items.

Consequently, the manufacturer's income from the certification fee diminishes. The manufacturer raises the certification fee to offset this loss, encouraging the remanufacturer to reduce production. This ultimately lowers the profits for both the manufacturer and the remanufacturer. Secondly, a high new (or remanufactured) production cost significantly increases the production quantities of remanufactured (or new) products. To counteract losses caused by rising production costs, the manufacturer reduces the certification fee to encourage remanufacturers to purchase more certifications. With an increase in remanufacturing costs, the output decreases. To secure higher profits, manufacturers might again reduce the certification fee to attract more remanufacturer purchases, creating a cyclical pattern.

Proposition 1

(1) The quantities of new medical products are decreasing in the government subsidy t and new medical product production $c_{N}$ in Scenario B, N, and C, but the decreasing rate in Scenario B is the largest, followed by Scenario N and Scenario C (i.e., $\frac{\partial q_{N}^{B}}{\partial t}<\frac{\partial q_{N}^{N}}{\partial t}<\frac{\partial q_{N}^{C}}{\partial t}<0,\frac{\partial q_{N}^{B}}{\partial c_{N}}<\frac{\partial q_{N}^{N}}{\partial c_{N}}<\frac{\partial q_{N}^{C}}{\partial c_{N}}<0$).
(2) The quantities of remanufactured medical products are increasing due to the government subsidy t and new medical products production $c_{N}$ in Scenario B, N, and C, but the increasing rate in Scenario B is the largest, followed by Scenario N and Scenario C (i.e., $\frac{\partial q_{R}^{B}}{\partial t}>\frac{\partial q_{R}^{N}}{\partial t}>\frac{\partial q_{R}^{C}}{\partial t}>0,\frac{\partial q_{R}^{B}}{\partial c_{N}}>\frac{\partial q_{R}^{N}}{\partial c_{N}}>\frac{\partial q_{R}^{C}}{\partial c_{N}}>0$).
(3) The quantities of the new (remanufactured) medical products are increasing (decreasing) in the remanufactured medical products' production $c_{R}$ in Scenario B, N, and C, but the increasing (decreasing) rate in Scenario B is the largest, followed by Scenario N and Scenario C (i.e., $\frac{\partial q_{N}^{B}}{\partial c_{R}}>\frac{\partial q_{N}^{N}}{\partial c_{R}}>\frac{\partial q_{N}^{C}}{\partial c_{R}}>0,\frac{\partial q_{R}^{B}}{\partial c_{R}}<\frac{\partial q_{R}^{N}}{\partial c_{R}}<\frac{\partial q_{R}^{C}}{\partial c_{R}}<0$).

Proposition 1 shows that government subsidies can augment the marginal profit of remanufactured medical products, stimulating their production. In Scenario B, this increase in remanufactured medical products yields more subsidy-related benefits for manufacturers. Consequently, this encourages them to curtail new medical product manufacturing significantly. In Scenario N, remanufactured medical products possess greater competitive advantages, which predisposes consumers towards these remanufactured medical products. This shifts the manufacturer's motivation away from creating new medical products. However, the rate of decrease in this scenario is less than in Scenario C. In Scenario C, although there's a reduction in new medical product manufacturing, the certification fee paid by remanufacturers helps offset this loss. Additionally, the heightened cost of newly produced medical products results in a cutback of these products. This further bolsters the production of remanufactured medical products, as the certification fee, once again, compensates for any loss resulting from reduced new medical product output. Therefore, the rate of decrease in Scenario N is less than that in Scenario C. Simultaneously, with the cost of remanufactured medical products rising, manufacturers, in their quest for increased profits, are likely to abandon the production of remanufactured medical products in favor of new ones. Correspondingly, to mitigate losses, remanufacturers may opt for reduced production. Moreover, the rate of decrease in Scenario N exceeds that in Scenario C. This is because, in Scenario C, remanufactured medical products can enhance consumer preference by paying certification fees to manufacturers. This effectively counterbalances any loss engendered by a surge in production costs.

Proposition 2

(1) The total quantities of the new and remanufactured medical products in Scenario N and C are increasing in t, and there is no effect in Scenario B (i.e., $\frac{\partial q_{N}^{B}+\partial q_{R}^{B}}{\partial t}=0,\frac{\partial q_{N}^{N}+\partial q_{R}^{N}}{\partial t}>\;\frac{\partial q_{N}^{C}+\partial q_{R}^{C}}{\partial t}>0$).
(2) The total quantities of the new and remanufactured medical products in Scenarios N and C are decreasing in $c_{R}$, there is no effect in Scenario B (i.e., $\frac{\partial q_{N}^{B}+\partial q_{R}^{B}}{\partial c_{R}}=0,\frac{\partial q_{N}^{N}+\partial q_{R}^{N}}{\partial c_{R}}<\;\frac{\partial q_{N}^{C}+\partial q_{R}^{C}}{\partial c_{R}}<0$).
(3) The total quantities of the new and remanufactured medical products in Scenario B, N, and C are decreasing in $c_{N}$ (i.e., $\frac{\partial q_{N}^{B}+\partial q_{R}^{B}}{\partial c_{N}}<\frac{\partial q_{N}^{N}+\partial q_{R}^{N}}{\partial c_{N}}<\frac{\partial q_{N}^{C}+\partial q_{R}^{C}}{\partial c_{N}}<0$).

Proposition 2 delineates that government subsidies effectively stimulate the total production of new and remanufactured medical products in Scenarios N and C. Particularly in Scenario N, the relationship between the increase in government subsidies and the marginal income of remanufactured medical products is direct. In other words, the greater the subsidy increase, the higher the marginal income of remanufactured medical products, thus increasing total output. On the other hand, in Scenario B, manufacturers produce new medical products and remanufactured ones. In this case, increasing government subsidies does not impact the total production of medical products. This is because when manufacturers augment the production of remanufactured medical products, they proportionally decrease the production of new ones. Moreover, though subsidies enhance the marginal revenue of remanufactured medical products, the remanufacturer in Scenario C incurs a greater certification fee paid to the manufacturer. Consequently, the rate of increase in total medical product quantity is not as high as in Scenario N. As the production cost of remanufactured products escalates, the total output of new and remanufactured medical products in Scenarios N and C declines. This occurs because the increase in new product manufacture does not compensate for the drop in remanufactured product output. Additionally, in Scenario B, manufacturers counteract the increased cost by expanding the production of new medical products while curtailing that of remanufactured ones, leaving the total product count unaffected. Concurrently, increased production costs of newly manufactured products decrease the total output of new and remanufactured medical products across all scenarios B, N, and C. The rationale behind this trend is that the rise in remanufactured product output does not offset the loss incurred in producing new products.

Corollary 4 . In the feasible range, the equilibrium outcomes satisfy: the total quantities of the new and remanufactured medical products in Scenario C are higher than in Scenario N, followed by in Scenario B when $0<m^{a}<m<1$.

$$\text{Where}\;m^{a}=\frac{5+4t+8\beta +8t\beta -c_{N}-4c_{R}-8\beta c_{R}}{10+16\beta }$$

Corollary 4 demonstrates that the enhanced brand image of medical products motivates remanufacturers to increase production. Specifically, remanufacturers augment their perceived advantage in medical product remanufacturing by remitting certification fees to the original manufacturers, thereby bolstering their production incentives. It follows logically that when medical products possess a strong brand image, the aggregate quantities of new and remanufactured medical products are substantial, particularly in Scenario C.

Proposition 3 Within the feasible range of production for new medical products, a comparative analysis of the manufacturer's profit across three distinct strategies reveals that the profit accrued under Strategy C consistently surpasses that under the alternative scenarios.

Proposition 3 indicates that as the number of remanufactured medical products increases, it instigates intensified competition with newly produced medical products within the downstream market. This competition consequently diminishes the manufacturer's profit derived from producing new products. While the escalation in the quantity of remanufactured medical products fosters vigorous competition with new items in the downstream market, under Strategy C, the remanufacturer compensates the manufacturer by paying a certification fee. The increase in these fees correlates with an augmentation in the manufacturer's marginal profit.

Proposition 4 Within the feasible range of production for remanufactured medical products, comparing the remanufacturer's profit across three distinct strategies reveals that the profit realized under Strategy B consistently exceeds that obtained under the other scenarios.

Proposition 4 elucidates that the remanufacturer consistently derives greater profit from Strategy B, wherein manufacturers also produce remanufactured medical products. Since consumers' trust in brands eliminates brand differentiation among remanufactured medical products, an increase in the production of such items enhances the profit scale for manufacturers.

Conversely, with the rise in remanufactured medical products, brand differences emerge between those produced by the remanufacturer under Strategy N and those produced by the original manufacturer. As a result, the profit derived from producing remanufactured medical products under Strategy N is less than that realized under Strategy B. Furthermore, under Strategy C, the payment of a certification fee by the remanufacturer to the brand manufacturer ensures that no brand differentiation occurs between remanufactured medical products and those produced by the original manufacturer. Nevertheless, the high certification fee incurs specific losses for remanufacturers and amplifies their marginal profits. Therefore, in the context of the production of remanufactured medical products, the remanufacturer consistently reaps greater profits under Strategy B.

Table 3, Table 4 illustrate the impact of government subsidies on the production of new and refurbished products, as well as the overall profits of the supply chain. When per-unit costs for sustainable production are low, a vertical analysis reveals that under Strategy B, even with minimal government subsidies, manufacturers promptly switch to remanufacturing medical products, abandoning new product production. Conversely, in Strategy N, manufacturers begin remanufacturing only when subsidies reach a certain threshold. As government subsidies increase, the entire supply chain's profits decrease under Strategy N, while profits rise under the other strategies. In scenarios where per-unit sustainable production costs are high, a similar vertical comparison in Strategy B shows that manufacturers start remanufacturing medical products once government subsidies reach a specific level. In Strategy N, remanufacturing commences only after subsidies increase sufficiently. The trend in overall supply chain profits mirrors that observed at lower sustainable production costs. However, when government subsidies are relatively low, the supply chain profits in the Strategy N scenario surpass those of other strategies.

**Table 3:** Effect of government subsidy t on selling quantity q when per-unit sustainability production cost β is low (whenthecostβ=0.15). When the unit cost of sustainable production β is low, the outcomes vary by scenario. In Scenario B, the manufacturer shifts from new product production to remanufacturing regardless of government subsidy t. In Scenario N, increasing government subsidies t leads to remanufactured product production. In Scenario C, remanufactured products are produced regardless of subsidies.

t	Strategy B			Strategy N			Strategy C		
	$q_{N}^{B}$	$q_{R}^{B}$	$\pi^{B}$	$q_{N}^{N}$	$q_{R}^{N}$	$\pi^{N}$	$q_{N}^{C}$	$q_{R}^{C}$	$\pi^{C}$
0.05	-	0.53	0.36	0.44	-	0.36	0.37	0.05	0.33
0.1	-	0.70	0.39	0.43	-	0.34	0.37	0.07	0.33
0.15	-	0.87	0.43	0.41	-	0.33	0.36	0.08	0.34
0.2	-	1.03	0.48	0.40	0.01	0.32	0.35	0.10	0.35
0.25	-	1.20	0.54	0.38	0.03	0.31	0.34	0.12	0.36
0.3	-	1.37	0.60	0.37	0.06	0.30	0.33	0.13	0.37
0.35	-	1.53	0.67	0.36	0.09	0.30	0.33	0.15	0.38
0.4	-	1.70	0.75	0.34	0.12	0.29	0.32	0.16	0.39
0.45	-	1.87	0.84	0.33	0.14	0.29	0.31	0.18	0.41
0.5	-	2.03	0.94	0.31	0.17	0.29	0.30	0.20	0.42

Scenario B: Combined Production of New and Refurbished Products Scenario

Scenario N: Independent Production of New and Refurbished Products by Manufacturers and Remanufacturers Scenario

Scenario C: Independent Production of New and Refurbished Products, Including a Remanufacturer Certification Fee

**Table 4:** Effect of government subsidy t on selling quantity q when per-unit sustainability production cost β is high (whenthecostβ=0.45). When the unit cost of sustainable production β is high, the outcomes vary by scenario. In Scenario B, as the government subsidy t increases, the manufacturer gradually abandons new product production in favor of remanufacturing. In Scenario N, remanufactured product production begins only after a substantial increase in government subsidy t. In Scenario C, remanufactured products are produced regardless of subsidy levels.

t	Strategy B			Strategy N			Strategy C		
	$q_{N}^{B}$	$q_{R}^{B}$	$\pi^{B}$	$q_{N}^{N}$	$q_{R}^{N}$	$\pi^{N}$	$q_{N}^{C}$	$q_{R}^{C}$	$\pi^{C}$
0.05	0.22	0.18	0.33	0.43	-	0.35	0.38	0.04	0.32
0.1	0.17	0.23	0.34	0.42	-	0.34	0.38	0.05	0.33
0.15	0.11	0.29	0.36	0.41	-	0.33	0.37	0.06	0.33
0.2	0.06	0.34	0.37	0.40	0.00	0.32	0.36	0.07	0.34
0.25	0.00	0.40	0.39	0.39	0.03	0.31	0.36	0.08	0.35
0.3	-	0.46	0.41	0.38	0.05	0.31	0.35	0.10	0.35
0.35	-	0.51	0.44	0.37	0.07	0.30	0.35	0.11	0.36
0.4	-	0.57	0.46	0.36	0.09	0.30	0.34	0.12	0.37
0.45	-	0.62	0.49	0.35	0.11	0.30	0.33	0.13	0.38
0.5	-	0.68	0.53	0.34	0.13	0.30	0.33	0.14	0.39

Scenario B: Combined Production of New and Refurbished Products Scenario

Scenario N: Independent Production of New and Refurbished Products by Manufacturers and Remanufacturers Scenario

Scenario C: Independent Production of New and Refurbished Products, Including a Remanufacturer Certification Fee

**Fig. 2:**
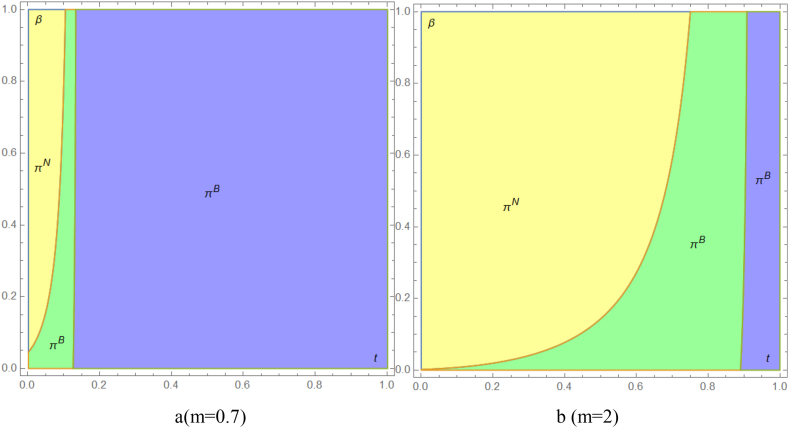
The entire supply chain preference strategy. The entire product supply chain favors Strategy N when there is no government subsidy. When both government subsidies t and unit sustainable production costs β are low, the supply chain primarily favors Strategy B. As government subsidies t and unit sustainable production costs β increase, the supply chain becomes more inclined toward Strategy B. Additionally, the disadvantage of remanufactured products, m, amplifies this trend. Scenario B involves the joint production of new and refurbished products, while Scenario N represents manufacturers' and remanufacturers' independent production.

Drawing upon the research by Yu et al. [38] and Niu et al. [27], this study sets the parameters $c_{N}=0.2$ and $c_{R}=0.09$ to examine the impact of varying government subsidies t and per-unit sustainability production costs β on the selection of production strategies within supply chains. As depicted in Fig. 2, under conditions of exceptionally low government subsidies and minimal per-unit sustainability production costs, the supply chain predominantly favors Strategy B, followed by Strategy N. This trend arises because negligible government subsidies offer limited appeal to remanufacturers. Concurrently, the low sustainability production costs associated with remanufactured products do not confer a significant competitive edge. However, as governmental environmental consciousness intensifies, remanufactured products begin to exhibit a competitive advantage, significantly encouraging remanufacturers. In this scenario, there is no market encroachment on new products. Moreover, this approach compensates for the heightened per-unit sustainability production costs of remanufactured items. To foster a mutually beneficial outcome, supply chains are more inclined to adopt Strategy N, with Strategy B as a secondary preference. Conversely, in situations where government subsidies are exceptionally high, Strategy B emerges as the more viable option for the entire supply chain. High subsidies lead to an increased production of remanufactured products, potentially creating a predatory impact on the market for new products and culminating in a lose-lose scenario. Additionally, this becomes more evident when the disadvantages of remanufactured medical products are more significant (Fig. 2 a and b).

We analyze the government's maximized utility function (social welfare), which illuminates the motivation behind government intervention and the strategic basis of government subsidy policy. The government may encourage remanufacturers to produce remanufactured medical products through government subsidies. In our model, the government maximizes its objective function by subsidizing remanufacturers based on the quantity of remanufactured medical products produced. Thus, the government aims to spend money on subsidies and social welfare.

(8) $$\begin{array}{c} \pi_{g}^{j}={SW}^{j}-tq_{R}^{j} \end{array}$$

In the above equation (8), the first term is social welfare, and the second is the government's subsidy expenditure. Where t is the government subsidy per unit of remanufactured medical product. Following Singh and Vives [39] and Chen et al. [35], the first term social welfare can be expressed as equation (9)

(9) $$\begin{array}{c} {SW}^{j}=\pi_{M}^{j}+\pi_{R}^{j}+{CS}^{j} \end{array}$$

Where $\pi_{R}^{j}$ is the incremental increase in social welfare from remanufactured medical products. ${CS}^{j}$ is Consumer surplus, which is commonly used in the literature by Singh and Vives [39], can be characterized as equation (10)

(10) $$\begin{array}{c} {CS}^{j}=\frac{1}{2}{\left({q_{N}^{j}+q}_{R}^{j}\right)}^{2} \end{array}$$

Then, the government maximizes objective function under scenarios B, N, and C, respectively (Equations (11), (12), (13)),

(11) $$\begin{array}{c} \pi_{g}^{B}=\pi_{M}^{B}+\pi_{R}^{B}+\frac{1}{2}{\left(q_{N}^{B}+q_{R}^{B}\right)}^{2}-tq_{R}^{B} \end{array}$$

(12) $$\begin{array}{c} \pi_{g}^{N}={\pi_{M}^{N}+\pi }_{R}^{N}+\frac{1}{2}{\left(q_{N}^{N}+q_{R}^{N}\right)}^{2}-tq_{R}^{N} \end{array}$$

(13) $$\begin{array}{c} \pi_{g}^{C}=\pi_{M}^{C}+\pi_{R}^{C}+\frac{1}{2}{\left(q_{N}^{C}+q_{R}^{C}\right)}^{2}-tq_{R}^{C} \end{array}$$

By substituting the optimal output $q_{N}^{j}$ and $q_{R}^{j}$ into $\pi_{g}^{j}$, we can get equations (14), (15), (16)

(14) $$\begin{array}{c} \pi_{g}^{B}=\frac{4\beta -t^{2}+\left(1+4\beta \right)c_{N}^{2}+c_{R}^{2}-2c_{N}\left(4\beta +c_{R}\right)}{4\beta } \end{array}$$

(15) $$\pi_{g}^{N}=\frac{\begin{array}{c} 2{\left(3+4\beta \right)}^{2}{\left(1-c_{N}\right)}^{2}-8t\left(3+4\beta \right)\left(1-2m+2t+c_{N}-2c_{R}\right)\\ +8\left(1+\beta \right){\left(1-2m+2t+c_{N}-2c_{R}\right)}^{2}+8{\left(1+m-t+2\beta -2\left(1+\beta \right)c_{N}+c_{R}\right)}^{2}\\ +{\left(-7+2m-2t-8\beta +\left(5+8\beta \right)c_{N}+2c_{R}\right)}^{2} \end{array}}{8{\left(3+4\beta \right)}^{2}}$$

(16) $$\pi_{g}^{C}=\frac{\begin{array}{c} 2t\left(5+8\beta \right)-t^{2}\left(1+8\beta \right)+2{\left(5+8\beta \right)}^{2}+\left[59+8\beta \left(21+16\beta \right)\right]c_{N}^{2}\\ +c_{N}(8t-2\left(5+8\beta \right)\left[9+16\beta )-4\left(7+8\beta \right)c_{R}\right]\\ -2c_{R}\left[(5+9t+8\left(1+t\right)\beta \right]+{c_{R}}^{2}\left(19+24\beta \right) \end{array}}{2{\left(5+8\beta \right)}^{2}}$$

The first-order condition for deciding an optimal t under scenario B, N, and C, respectively,

$$\frac{\partial \pi_{g}^{B}}{\partial t}=-\frac{t}{2\beta }$$

$$\frac{\partial \pi_{g}^{N}}{\partial t}=\frac{5-10m-2t-16t\beta +5c_{N}-10c_{R}}{2{\left(3+4\beta \right)}^{2}}$$

$$\frac{\partial \pi_{g}^{C}}{\partial t}=\frac{5-t+8\left(1-t\right)\beta +4c_{N}-\left(9+8\beta \right)c_{R}}{{\left(5+8\beta \right)}^{2}}$$

Thus, we can obtain the optimal t under scenarios N and C, respectively,

$$t^{N*}=\frac{5\left(1-2m+c_{N}-2c_{R}\right)}{2+16\beta }$$

$$t^{C*}=\frac{5+8\beta +4c_{N}-\left(9+8\beta \right)c_{R}}{1+8\beta }$$

Corollary 5 .

(1) Under scenario B, government subsidies have a negative impact on social welfare.
(2) under scenario N, there exists an optimal $t^{N*}$ when $t^{C}<t^{C*}\text{,}$ where overall social welfare increases with government subsidies; otherwise $\pi_{g}^{C}$ decreases in $t^{C}\text{.}$
(3) Under scenario C, there exists an optimum $t^{C*}\text{,}$ where overall social welfare increases with government subsidies when $t^{C}<t^{C*}$; otherwise $\pi_{g}^{C}$ decreases in $t^{C}\text{.}$

Corollary 5 suggests that increasing government subsidies may positively or negatively affect overall social welfare. The government does not negatively affect social welfare for scenario B firms even if it does not subsidize. For firms in scenarios N and C, the government should increase government subsidies while preventing "capital transfers" from the firms, thereby increasing overall social welfare.

**Fig.3:**
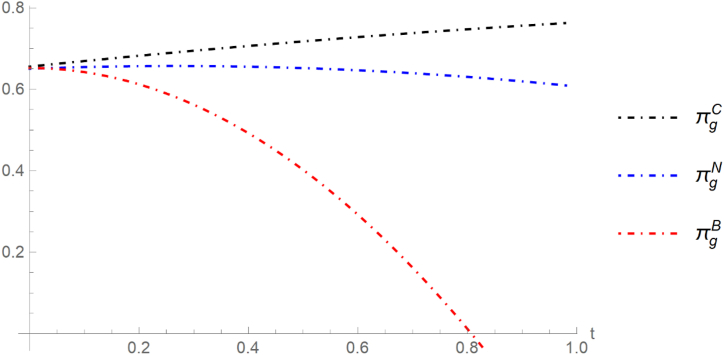
Social welfare in different scenarios. As government subsidies t increase, overall social welfare shows different trends under each strategy. Under Strategy C, social welfare consistently increases. Under Strategy N, social welfare increases initially but then decreases. Under Strategy B, social welfare continually decreases. Scenario B involves the joint production of new and refurbished products. Scenario N represents manufacturers' and remanufacturers' independent production of new and refurbished products. Scenario C includes the independent production of new and refurbished products, accounting for remanufacturer certification fees.

By using the numerical simulation method, we assume $c_{N}=0.2,c_{R}=0.09,m=0.35,\beta =0.25$. Fig. 3 shows that with the increase of government subsidies, under scenario C, the whole social welfare is always in the highest position. In other words, strategy C can maximize the whole social welfare.

## 4 Extensions

### 4.1 Sequential Quantity Determination: Remanufacturer's Decision Following Manufacturer's

In the three delineated strategies, the scenario where the remanufacturer decides the quantity of its refurbished medical products only after receiving the order number from the manufacturer is denoted with the superscript 'R'. Under this condition, we obtain the equilibrium outcome as presented in Lemma 4.

Lemma 4 The manufacturer and remanufacturer's optimal decisions.

1) Under scenario B: $q_{N1}^{BR}=\frac{1-c_{N}}{2},q_{N}^{BR}=\frac{\beta -t-\left(1+\beta \right)c_{N}+c_{R}}{2\beta }$ and $q_{R}^{BR}=\frac{t+c_{N}-c_{R}}{2\beta }\text{,}$ respectively. Correspondingly, the profits of the manufacturer are $\pi_{M}^{BR}=\frac{t^{2}+2\beta +c_{N}\left(2t-4\beta +c_{N}+2\beta c_{N}\right)-2\left(t+c_{N}\right)c_{R}+c_{R}^{2}}{4\beta }$.
2) Under scenario N: $q_{N1}^{NR}=\frac{1-c_{N}}{2}\text{,}$ $q_{N}^{NR}=\frac{1+m-t+2\beta -2\left(1+\beta \right)c_{N}+c_{R}}{2+4\beta }\text{,}$ and $q_{R}^{NR}=\frac{1-m+t-c_{R}-\frac{1+m-t+2\beta -2\left(1+\beta \right)c_{N}+c_{R}}{2+4\beta }}{2\left(1+\beta \right)}\text{,}$ respectively. Correspondingly, the profits of the manufacturer and remanufacturer are $\pi_{M}^{NR}=\frac{3+m^{2}+\left(-2+t\right)t+10\beta -4t\beta +8\beta^{2}+m\left(2-2t+4\beta \right)+2\left(1+\beta \right)\left(3+4\beta \right)c_{N}^{2}+c_{R}\left(2+2m-2t+4\beta +c_{R}\right)-4\left(1+\beta \right)c_{N}\left(2+m-t+4\beta +c_{R}\right)}{8\left(1+\beta \right)\left(1+2\beta \right)}$ and $\pi_{R}^{NR}=\frac{{\begin{array}{c} (1-3m+3t+2\beta -4m\beta +4t\beta \end{array}}^{2}+2\left(1+\beta \right)c_{N}-\left(3+4\beta \right)c_{R})}{16\left(1+\beta \right){\left(1+2\beta \right)}^{2}}\text{,}$ respectively.
3) Under scenario C: the manufacturer and remanufacturer's optimal decisions are $q_{N1}^{CR}=\frac{1-c_{N}}{2}\text{,}$ $q_{N}^{CR}=\frac{1-t+2\beta -2\left(1+\beta \right)c_{N}+c_{R}}{2+4\beta }$ and $q_{R}^{CR}=\frac{1-f+t-c_{R}-\frac{1-t+2\beta -2\left(1+\beta \right)c_{N}+c_{R}}{2+4\beta }}{2\left(1+\beta \right)}\text{,}$ respectively. The certification fee $f^{R}=\frac{1+t-c_{R}}{2}\text{.}$ Correspondingly, the profits of the manufacturer and remanufacturer are $\pi_{M}^{CR}=\frac{\begin{array}{c} 2+t^{2}+4\beta +c_{N}\left(2\left(-2+t-4\beta \right)+\left(3+4\beta \right)c_{N}\right)-2\left(t+c_{N}\right)c_{R}+c_{R}^{2} \end{array}}{4+8\beta }$ and $\pi_{R}^{CR}=\frac{\left(1+\beta \right){\left(t+c_{N}-c_{R}\right)}^{2}}{4{\left(1+2\beta \right)}^{2}}\text{,}$ respectively.

Proposition 5

(1) The quantities of new medical products are decreasing due to the government subsidy t and new medical products production cost $c_{N}$ in Scenario B, N, and C, but the decreasing rate in Scenario B is the largest, The quantities of new medical products are decreasing in the government subsidy t, and new medical products production cost $c_{N}$ in Scenario B, N, and C, but the decreasing rate in Scenario B is the largest, while Scenarios N and C share an identical rate of decline (i.e., $\frac{\partial q_{N}^{BR}}{\partial t}<\frac{\partial q_{N}^{NR}}{\partial t}=\frac{\partial q_{N}^{CR}}{\partial t}<0,\frac{\partial q_{N}^{BR}}{\partial c_{N}}<\frac{\partial q_{N}^{NR}}{\partial c_{N}}=\frac{\partial q_{N}^{CR}}{\partial c_{N}}<0$).
(2) The quantities of remanufactured medical products are increasing due to the government subsidy t and new medical products' production cost $c_{N}$ in Scenario B, N, and C. As the government subsidy t increases, the rate of this increase is most significant in Scenario B, followed by Scenarios N and C. Concurrently, with the rising cost of new medical product production $c_{N}$, the increasing rate remains highest in Scenario B, while Scenarios N and C exhibit identical rates of increase (i.e., $\frac{\partial q_{R}^{BR}}{\partial t}>\frac{\partial q_{R}^{NR}}{\partial t}>\frac{\partial q_{R}^{CR}}{\partial t}>0,\frac{\partial q_{R}^{BR}}{\partial c_{N}}>\frac{\partial q_{R}^{NR}}{\partial c_{N}}=\frac{\partial q_{R}^{CR}}{\partial c_{N}}>0$).
(3) The quantities of the new (remanufactured) medical products are increasing (decreasing) in the remanufactured medical products' production $c_{R}$ in Scenario B, N, and C, but the increasing (decreasing) rate in Scenario B is the largest, followed by Scenario C and Scenario N (i.e., $\frac{\partial q_{N}^{BR}}{\partial c_{R}}>\frac{\partial q_{N}^{CR}}{\partial c_{R}}>\frac{\partial q_{N}^{NR}}{\partial c_{R}}>0,\frac{\partial q_{R}^{BR}}{\partial c_{R}}<\frac{\partial q_{R}^{CR}}{\partial c_{R}}<\frac{\partial q_{R}^{NR}}{\partial c_{R}}<0$).

$$\text{Here}\;\frac{\partial q_{N}^{BR}}{\partial t}=\frac{-1}{2\beta },\frac{\partial q_{N}^{NR}}{\partial t}=\frac{-1}{2+4\beta },\frac{\partial q_{N}^{CR}}{\partial t}=\frac{-1}{2+4\beta }\text{;}$$

$$\frac{\partial q_{N}^{BR}}{\partial c_{N}}=\frac{-1-\beta }{2\beta },\frac{\partial q_{N}^{NR}}{\partial c_{N}}=\frac{-1-\beta }{1+2\beta },\frac{\partial q_{N}^{CR}}{\partial c_{N}}=\frac{-1-\beta }{1+2\beta }\text{;}$$

$$\frac{\partial q_{R}^{BR}}{\partial t}=\frac{1}{2\beta },\frac{\partial q_{R}^{NR}}{\partial t}=\frac{3+4\beta }{4+12\beta +8\beta^{2}},\frac{\partial q_{R}^{CR}}{\partial t}=\frac{1}{2+4\beta }\text{;}$$

$$\frac{\partial q_{R}^{BR}}{\partial c_{N}}=\frac{1}{2\beta },\frac{\partial q_{R}^{NR}}{\partial c_{N}}=\frac{1}{2+4\beta },\frac{\partial q_{R}^{CR}}{\partial c_{N}}=\frac{1}{2+4\beta }\text{;}$$

$$\frac{\partial q_{N}^{BR}}{\partial c_{R}}=\frac{1}{2\beta },\frac{\partial q_{N}^{NR}}{\partial c_{R}}=\frac{1}{3+4\beta },\frac{\partial q_{N}^{CR}}{\partial c_{R}}=\frac{1}{2+4\beta }\text{;}$$

$$\frac{\partial q_{R}^{BR}}{\partial c_{R}}=\frac{-1}{2\beta },\frac{\partial q_{R}^{NR}}{\partial c_{R}}=\frac{-2}{3+4\beta },\frac{\partial q_{R}^{CR}}{\partial c_{R}}=\frac{-1}{2+4\beta }\text{.}$$

Lemma 4 elucidates the equilibrium outcomes across varying strategies. A comparative analysis of Proposition 1 and Proposition 5 reveals a consistent influence of government subsidies and product production costs on the characteristics of new and refurbished products, affirming the robustness of our findings in the principal analysis. Notably, under Scenario C (Certification), these factors exert a more pronounced effect on the strategic decisions of supply chain participants. This heightened impact is attributed to the manufacturer's first-mover advantage, which enables the remanufacturer to leverage increased subsidies by augmenting the output of remanufactured products. Consequently, the decision-making process becomes increasingly sensitive to variations in remanufacturing costs.

**Fig.4:**
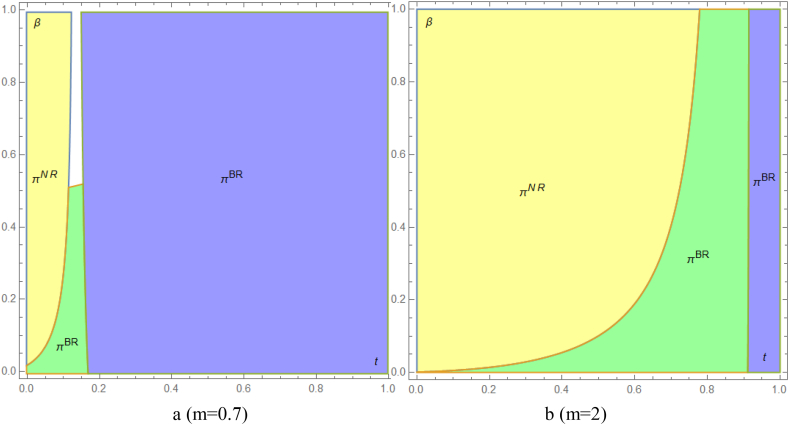
The entire supply chain preference strategy. In the manufacturer-first decision model, the supply chain favors Strategy NR without government subsidies t. When subsidies and unit sustainable production costs β are low, joint production strategies are preferred. As subsidies t and costs β increase, Strategy BR becomes more favored, with the disadvantage of remanufactured products m reinforcing this trend. Scenario NR represents joint production in the manufacturer's decision-making model. Scenario N involves independent production by manufacturers and remanufacturers. Scenario C includes manufacturer-first independent production and remanufacturer certification fees.

Fig. 4 demonstrates that the overall preference scenario of the supply chain remains consistent across the three strategies, thereby confirming the robustness of our results presented in the main context. The primary variation arises when the disadvantages of remanufactured medical products are minimal. In such cases, if government subsidies are also reduced, the entire supply chain will opt for Strategy B, provided that the per-unit cost of sustainable production remains reasonably low (Fig. 4 a and b).

## 1 Conclusion and Managerial Insights

The pandemic has led to a significant shortage of medical supplies, resulting in numerous challenges within the medical sector. To promote the sustainable use of these supplies, governments have introduced incentive-based regulations, such as subsidies for remanufacturing medical products. In response to these incentives, existing manufacturers have started producing remanufactured medical goods, and new manufacturers have also entered the remanufacturing sector. The influx of these remanufacturing entities undoubtedly presents challenges for traditional manufacturers. However, suppose a cooperative and competitive relationship between original manufacturers and remanufacturers is maintained over the long term. In that case, it can enhance the sustainability of the entire medical production system, thereby alleviating supply shortages.

In our study, we devised three production game models to address medical product shortages: Scenario B, where manufacturers produce both new and refurbished products; Scenario N, where manufacturers produce new products and remanufacturers handle refurbished ones; and Scenario C, akin to Scenario N but with remanufacturers paying a certification fee to original manufacturers to minimize brand differentiation. Our findings suggest that, during a pandemic, the Scenario C (Certification) strategy yields the highest total production of medical products. This approach is most effective in mitigating medical product shortages, enhancing the sustainability of the entire medical production system, and alleviating supply chain disruptions. Furthermore, under the Scenario C (Certification) strategy, even in the absence of government subsidies, manufacturers can offset potential losses through the certification fees paid by remanufacturers, rendering this strategy more economically viable.

Our research offers significant insights for medical product manufacturers, remanufacturers, and policymakers. For manufacturers, while government subsidies for remanufactured products enhance the production of such items, they might not significantly influence the output of new medical products under Scenario B. As these subsidies rise, there's an observable increase in refurbished product numbers and a corresponding decrease in new products. However, it's crucial to highlight that under both Scenario N and Scenario C, the supply chain's overall quantity of medical products sees an uptick. This information serves as a theoretical guide to address medical product shortages during emergencies, stabilizing the broader societal implications of such shortages.

### 5.1 Managerial Insights

This study synthesizes our prior research and analysis to inform the development of environmental policies for governments overseeing medical supply manufacturing and remanufacturing. The following points encapsulate key management implications derived from the proposed model:

1. Managers in medical product manufacturing should recognize the substantial benefits of a joint remanufacturing strategy. This approach notably enhances the stability of the medical product supply chain during crises and aids in environmental conservation. Notably, under specific conditions, such as when government subsidies are minimal, the supply chain resilience offered by Strategy N surpasses that of alternative strategies. Consequently, government entities may show a preference for the adoption of Strategy N-focused alliances. However, if managers prioritize solely the manufacturer's profit, Strategy C is more advantageous.
2. Governments can fine-tune subsidy rates for remanufactured products to optimize supply chain resilience and environmental conservation. Nonetheless, a substantial increase in subsidies, while boosting overall supply chain profitability, imposes a greater financial burden on the government. This necessitates a prudent cap on subsidy rates.
3. In pursuit of an optimal balance among social, environmental, and economic outcomes, Strategy C is the most productive for the entire supply chain. Meanwhile, Strategy C is deemed superior for manufacturer-centric objectives. This paper introduces a game theory model, incorporating government subsidies and cooperative channels to foster strategic alliances and coordination within the medical product remanufacturing supply chain. The model is a vital guide for government policymakers and industry managers in advocating sustainable industry practices.

### 5.2 Limitations and Future Research

This study acknowledges several limitations, highlighting areas for future research. Firstly, the research does not account for the variability in product quality, basing its theoretical conclusions on consistent quality. However, in the medical sector, the quality of refurbished products is often unpredictable. This uncertainty can exacerbate the perceived inferiority of remanufactured products, diminishing consumer demand. Consequently, manufacturers may be compelled to reduce prices, potentially harming market competition. Future research should thoroughly address the issue of quality variability. Secondly, the reliance on government subsidies is another concern, especially considering potential budgetary constraints. In a medical emergency with widespread economic repercussions, governments may reduce or eliminate these subsidies, adversely affecting the production of remanufactured medical products. This necessitates the exploration of effective regulatory frameworks that are not dependent on government subsidies. Thirdly, the current study's focus on government subsidies and production costs in the recycling and manufacturing processes of remanufactured medical products overlooks the aspect of production uncertainty. This represents a critical area for future investigation. Fourthly, the paper does not consider the differentiation between new and refurbished products in terms of the degree of consumer audience, and the uncertainty of refurbished products should be considered to address the differentiation between new and refurbished products in future research. Lastly, the potential for corruption in the recycling process of remanufactured medical products warrants further examination. This aspect is crucial for ensuring the integrity and efficacy of the recycling process and thus demands attention in subsequent research.

## Ethics approval

This study required no ethical approval.

## Data Availability Statement

This paper belongs to mathematical derivation which does not involve data availability.

## CRediT authorship contribution statement

**Yang Bai:** Writing – original draft, Visualization, Validation, Software, Methodology, Investigation, Conceptualization. **Yanjing Liu:** Writing – review & editing, Supervision, Software. **Shichao Han:** Writing – review & editing, Methodology, Investigation. **Wenqi Song:** Writing – original draft, Visualization, Formal analysis, Conceptualization.

## Declaration of competing interest

The authors declare that they have no known competing financial interests or personal relationships that could have appeared to influence the work reported in this paper.

## Appendix

### Proof of Lemma 1

Take the first-order conditions with respect to $q_{N}^{B}$ and $q_{R}^{B}$ to maximize the manufacturer's value function in the second period, given

$$\frac{\partial \pi_{M2}^{B}}{\partial q_{N}^{B}}=1-c_{N}-2q_{N}^{B}-2q_{R}^{B}=0$$

$$\frac{\partial \pi_{M2}^{B}}{\partial q_{R}^{B}}=1+t-c_{R}-2q_{N}^{B}-q_{R}^{B}+\left(-1-\beta \right)q_{R}^{B}-\beta q_{R}^{B}=0$$

Solving $q_{N}^{B}$ and $q_{R}^{B}$ simultaneously, we can easily obtain

$$q_{N}^{B}=\frac{\beta -t-\left(1+\beta \right)c_{N}+c_{R}}{2\beta }$$

$$q_{R}^{B}=\frac{t+c_{N}-c_{R}}{2\beta }$$

Take the first-order conditions with respect to $q_{N1}^{B}$ to maximize the manufacturer's value function in the first period, given

$$q_{N1}^{B}=1-c_{N}-2q_{N1}^{B}=0$$

Solving $q_{N1}^{B}$ we can easily obtain

$$q_{N1}^{B}=\frac{1}{2}\left(1-c_{N}\right)$$

Substituting $q_{N}^{B},q_{R}^{B}\text{,}$ and $q_{N1}^{B}$ into $\pi_{M}^{B}\text{,}$ then we have

$$\pi_{M}^{B}=\frac{t^{2}+2\beta +c_{N}\left(2t-4\beta +c_{N}+2\beta c_{N}\right)-2\left(t+c_{N}\right)c_{R}+c_{R}^{2}}{4\beta }$$

### Proof of Lemma 2

Take the first-order conditions with respect to $q_{N}^{N}$ and $q_{R}^{N}$ to maximize the manufacturer and remanufacturer's value function in the second period, respectively, given

$$\frac{\partial \pi_{M2}^{N}}{\partial q_{N}^{N}}=1-c_{N}-2q_{N}^{N}-q_{R}^{N}=0$$

$$\frac{\partial \pi_{R}^{N}}{\partial q_{R}^{N}}=1-m+t-c_{R}-q_{N}^{N}-q_{R}^{N}+\left(-1-\beta \right)q_{R}^{N}-\beta q_{R}^{N}=0$$

Solving $q_{N}^{N}$ and $q_{R}^{N}$ simultaneously, we can easily obtain

$$q_{N}^{N}=\frac{1+m-t+2\beta -2\left(1+\beta \right)c_{N}+c_{R}}{3+4\beta }$$

$$q_{R}^{N}=\frac{1-2m+2t+c_{N}-2c_{R}}{3+4\beta }$$

Take the first-order conditions with respect to $q_{N1}^{N}$ to maximize the manufacturer's value function in the first period, given

$$q_{N1}^{N}=1-c_{N}-2q_{N1}^{N}=0$$

Solving $q_{N1}$ we can easily obtain

$$q_{N1}^{N}=\frac{1}{2}\left(1-c_{N}\right)$$

Substituting $q_{N}^{N},q_{R}^{N}\text{,}$ and $q_{N1}^{N}$ into $\pi_{M}^{B}$, then we have

$$\pi_{M}^{N}=\frac{1}{4}{\left(-1+c_{N}\right)}^{2}+\frac{{\left(1+m-t+2\beta -2\left(1+\beta \right)c_{N}+c_{R}\right)}^{2}}{{\left(3+4\beta \right)}^{2}}$$

$$\pi_{R}^{N}=\frac{\left(1+\beta \right){\left(1-2m+2t+c_{N}-2c_{R}\right)}^{2}}{{\left(3+4\beta \right)}^{2}}$$

### Proof of Lemma 3

Take the first-order conditions with respect to $q_{N}^{C}$ and $q_{R}^{C}$ to maximize the manufacturer and remanufacturer's value function in the second period, respectively, given

$$\frac{\partial \pi_{M2}^{C}}{\partial q_{N}^{C}}=1-c_{N}-2q_{N}^{C}-q_{R}^{C}=0$$

$$\frac{\partial \pi_{R}^{C}}{\partial q_{R}^{C}}=1-f+t-c_{R}-q_{N}^{C}-q_{R}^{C}+\left(-1-\beta \right)q_{R}^{C}-\beta q_{R}^{C}=0$$

Solving $q_{N}^{C}$ and $q_{R}^{C}$ simultaneously, we can easily obtain

$$q_{N}^{C}=\frac{1+f-t+2\beta -2\left(1+\beta \right)c_{N}+c_{R}}{3+4\beta }$$

$$q_{R}^{C}=\frac{1-2f+2t+c_{N}-2c_{R}}{3+4\beta }$$

Take the first-order conditions with respect to $q_{N1}^{C}$ to maximize the manufacturer's value function in the first period, given

$$q_{N1}^{C}=1-c_{N}-2q_{N1}^{C}=0$$

Solving $q_{N1}^{C}$ we can easily obtain

$$q_{N1}^{C}=\frac{1}{2}\left(1-c_{N}\right)$$

Substituting $q_{N}^{C},q_{R}^{C}\text{,}$ and $q_{N1}^{C}$ into $\pi_{M}^{C}$ and take the first order condition with respect to f, then we have

$$f=\frac{5+4t+8\left(1+t\right)\beta -c_{N}-4\left(1+2\beta \right)c_{R}}{2\left(5+8\beta \right)}$$

Then we have

$$q_{N}^{C}=\frac{5-2t+8\beta -\left(7+8\beta \right)c_{N}+2c_{R}}{2\left(5+8\beta \right)}$$

$$q_{R}^{C}=\frac{2\left(t+c_{N}-c_{R}\right)}{5+8\beta }$$

$$f=\frac{5+4t+8\left(1+t\right)\beta -c_{N}-4\left(1+2\beta \right)c_{R}}{2\left(5+8\beta \right)}$$

$$\pi_{M}^{C}=\frac{5+2t^{2}+8\beta +c_{N}\left(2\left(-5+2t-8\beta \right)+\left(7+8\beta \right)c_{N}\right)-4\left(t+c_{N}\right)c_{R}+2c_{R}^{2}}{2\left(5+8\beta \right)}$$

$$\pi_{R}^{C}=\frac{4\left(1+\beta \right){\left(t+c_{N}-c_{R}\right)}^{2}}{{\left(5+8\beta \right)}^{2}}$$

### Proof of Proposition 2

Taking the first-order conditions of $q_{N}^{B}+q_{R}^{B}$, $q_{N}^{N}+q_{R}^{N}$, and $q_{N}^{C}+q_{R}^{C}$ with respect to t, we have $\frac{\partial q_{N}^{B}+\partial q_{R}^{B}}{\partial t}=0$

$$\frac{\partial q_{N}^{N}+\partial q_{R}^{N}}{\partial t}=\frac{1}{3+4\beta }>0$$

$$\frac{\partial q_{N}^{C}+\partial q_{R}^{C}}{\partial t}=\frac{1}{5+8\beta }>0$$

Similarly, we have

$$\frac{\partial q_{N}^{B}+\partial q_{R}^{B}}{\partial c_{R}}=0$$

$$\frac{\partial q_{N}^{N}+\partial q_{R}^{N}}{\partial c_{R}}=-\frac{1}{3+4\beta }<0$$

$$\frac{\partial q_{N}^{C}+\partial q_{R}^{C}}{\partial c_{R}}=-\frac{1}{5+8\beta }<0$$

And

$$\frac{\partial q_{N}^{B}+\partial q_{R}^{B}}{\partial c_{N}}=-\frac{1}{2}<0$$

$$\frac{\partial q_{N}^{N}+\partial q_{R}^{N}}{\partial c_{N}}=-\frac{1+2\beta }{3+4\beta }<0$$

$$\frac{\partial q_{N}^{C}+\partial q_{R}^{C}}{\partial c_{N}}=\frac{-3-8\beta }{2\left(5+8\beta \right)}<0$$

### Proof of Corollary 4

The total quantities of the new and remanufactured medical products in Scenario B are

$$q_{N+R}^{B}=1-c_{N}$$

The total quantities of the new and remanufactured medical products in Scenario N are

$$q_{N+R}^{N}=\frac{7-2m+2t+8\beta -\left(5+8\beta \right)c_{N}-2c_{R}}{6+8\beta }$$

The total quantities of the new and remanufactured medical products in Scenario C are

$$q_{N+R}^{C}=\frac{5+t+8\beta -4\left(1+2\beta \right)c_{N}-c_{R}}{5+8\beta }$$

It is easy to check $q_{N+R}^{N}-q_{N+R}^{B}=\frac{1-2m+2t+c_{N}-2c_{R}}{6+8\beta }>0\text{.}$ Next, we have to compare $q_{N+R}^{C}$ and $q_{N+R}^{N}$. Because $q_{N+R}^{C}-q_{N+R}^{N}=\frac{-5-4t-8\left(1+t\right)\beta +2m\left(5+8\beta \right)+c_{N}+\left(4+8\beta \right)c_{R}}{2\left(3+4\beta \right)\left(5+8\beta \right)}\text{,}$ when and only when $-5-4t-8\left(1+t\right)\beta +2m\left(5+8\beta \right)+c_{N}+\left(4+8\beta \right)c_{R}>0$,we can get $q_{N+R}^{C}>q_{N+R}^{N}\text{,}$ which means when $m^{a}=\frac{5+4t+8\beta +8t\beta -c_{N}-4c_{R}-8\beta c_{R}}{10+16\beta }$,Corollary 4 holds.

### Proof of Proposition 3

The difference in manufacturer's profits between $\pi_{M}^{C}$ and $\pi_{N}^{B}$ is

$$\pi_{M}^{C}-\pi_{N}^{B}=\frac{\left(t+c_{N}-c_{R}\right)\left(5+4\left(2+t\right)\beta -\left(5+4\beta \right)c_{N}-4\beta c_{R}\right)}{4\beta \left(5+8\beta \right)}$$

Based of $5+4\left(2+t\right)\beta -\left(5+4\beta \right)c_{N}-4\beta c_{R}>0$, we have $\pi_{M}^{C}-\pi_{N}^{B}>0\text{.}$

The difference of manufacturer's profits between $\pi_{M}^{C}$ and $\pi_{M}^{N}$ is

$$\pi_{M}^{C}-\pi_{M}^{N}=\frac{5+2t^{2}+8\beta +c_{N}\left(2\left(-5+2t-8\beta \right)+\left(7+8\beta \right)c_{N}\right)-4\left(t+c_{N}\right)c_{R}+2c_{R}^{2}}{2\left(5+8\beta \right)}-\frac{{\left(3+4\beta \right)}^{2}{\left(-1+c_{N}\right)}^{2}+4{\left(1+m-t+2\beta -2\left(1+\beta \right)c_{N}+c_{R}\right)}^{2}}{4{\left(3+4\beta \right)}^{2}}$$

Let $A=\frac{5+2t^{2}+8\beta +c_{N}\left(2\left(-5+2t-8\beta \right)+\left(7+8\beta \right)c_{N}\right)-4\left(t+c_{N}\right)c_{R}+2c_{R}^{2}}{2\left(5+8\beta \right)}-\frac{{\left(3+4\beta \right)}^{2}{\left(-1+c_{N}\right)}^{2}+4{\left(1+m-t+2\beta -2\left(1+\beta \right)c_{N}+c_{R}\right)}^{2}}{4{\left(3+4\beta \right)}^{2}}$, By calculating the first-order derivative of A(m,t) based on m, we have $\frac{\partial A\left(m,t\right)}{\partial m}=-\frac{2\left[1+m-t+2\beta -2\left(1+\beta \right)c_{N}+c_{R}\right]}{{\left(3+4\beta \right)}^{2}}<0\text{.}$ In such circumstances, based on $1+m-t+2\beta -2\left(1+\beta \right)c_{N}+c_{R}>0$, we can obtain A(m,t) increases with m. Obviously, we can derive that the minimum value of $B\;\left(t\right)=\mathrm{Min}\;{A\left(m,t\right)|}_{m=1}=\frac{\begin{array}{c} 5+2t^{2}+8\beta +c_{N}\left(2\left(-5+2t-8\beta \right)+\left(7+8\beta \right)c_{N}\right) \end{array}}{2\left(5+8\beta \right)}-\frac{\begin{array}{c} {\left(3+4\beta \right)}^{2}{\left(-1+c_{N}\right)}^{2}+4{\left(2-t+2\beta -2\left(1+\beta \right)c_{N}+c_{R}\right)}^{2} \end{array}}{4{\left(3+4\beta \right)}^{2}}$. By calculating the first-order derivative of B(t) based on t, we have $\frac{\partial B\;\left(t\right)}{\partial t}=\frac{4t+4c_{N}-4c_{R}}{2\left(5+8\beta \right)}+\frac{2\left(2-t+2\beta -2\left(1+\beta \right)c_{N}+c_{R}\right)}{{\left(3+4\beta \right)}^{2}}>0\text{.}$ Obviously, we can derive that $\mathrm{Min}\;{B\left(t\right)|}_{t=1}=\frac{2\left(c_{N}-c_{R}\right){\left(3+4\beta \right)}^{2}+2\left(2\left(1+\beta \right)\left(1-c_{N}\right)+c_{R}\right)\left(5+8\beta \right)}{\left(5+8\beta \right){\left(3+4\beta \right)}^{2}}>0\text{,}$ As a result, $\pi_{M}^{C}-\pi_{M}^{N}>0$ always hold.

Where $\pi_{N}^{B}$ is the manufacturer's profit from producing new products.

### Proof of Proposition 4

The difference in manufacturer's profits between $\pi_{R}^{C}$ and $\pi_{R}^{B}$ is

$$\pi_{R}^{C}-\pi_{R}^{B}=\frac{4\left(1+\beta \right){\left(t+c_{N}-c_{R}\right)}^{2}}{{\left(5+8\beta \right)}^{2}}-\frac{\left(1+t-c_{R}\right)\left(t+c_{N}-c_{R}\right)}{4\beta }$$

The difference in manufacturer's profits between $\pi_{R}^{N}$ and $\pi_{R}^{B}$ is

$$\pi_{R}^{N}-\pi_{R}^{B}=\frac{\left(1+\beta \right){\left(1-2m+2t+c_{N}-2c_{R}\right)}^{2}}{{\left(3+4\beta \right)}^{2}}-\frac{\left(1+t-c_{R}\right)\left(t+c_{N}-c_{R}\right)}{4\beta }$$

Let $E=\frac{4\left(1+\beta \right){\left(t+c_{N}-c_{R}\right)}^{2}}{{\left(5+8\beta \right)}^{2}}-\frac{\left(1+t-c_{R}\right)\left(t+c_{N}-c_{R}\right)}{4\beta }=\frac{\begin{array}{c} 16\beta \left(1+\beta \right){\left(t+c_{N}-c_{R}\right)}^{2}-\left(1+t-c_{R}\right)\left(t+c_{N}-c_{R}\right){\left(5+8\beta \right)}^{2} \end{array}}{4\beta {\left(5+8\beta \right)}^{2}}$, it is easy to see that the denominator is greater than zero. By calculating the first-order derivative of E(t,β) based on t, we have $F\left(t,\beta \right)=\frac{\partial E\left(t,\beta \right)}{\partial t}=32\beta \left(1+\beta \right)\left(t+c_{N}-c_{R}\right)-{\left(5+8\beta \right)}^{2}\left(t+c_{N}-c_{R}\right)-{\left(5+8\beta \right)}^{2}\left(1+t-c_{R}\right)$. Further, we have $G\left(t,\beta \right)=\frac{\partial F\left(t,\beta \right)}{\partial \beta }=-16\left(5+8t+8\beta +12t\beta +\left(3+4\beta \right)c_{N}-4\left(2+3\beta \right)c_{R}\right)\text{.}$ In such circumstances, we can obtain G(t,β)<0 always hold. Obviously, we can derive that the maximum value of $\mathrm{Max}\;{F\left(t,\beta \right)|}_{\beta =0}=-25\left(1+t-c_{R}\right)-25\left(t+c_{N}-c_{R}\right)<0$. Therefore, we can obtain E(t,β) decreases with t. Obviously, we can derive that $H\left(\beta \right)=\mathrm{Max}\;{E\left(t,\beta \right)|}_{t=1}=-{\left(5+8\beta \right)}^{2}\left(1-c_{R}\right)\left(c_{N}-c_{R}\right)+16\beta \left(1+\beta \right){\left(c_{N}-c_{R}\right)}^{2}\text{.}$ By calculating the first-order derivative of H(β) based on β, we have $\frac{\partial H\left(\beta \right)}{\partial \beta }=16\left(c_{N}-c_{R}\right)\left(-5-8\beta +\left(1+2\beta \right)c_{N}+\left(4+6\beta \right)c_{R}\right)<0\text{.}$ Then, we can obtain the maximum value of H(β) is $\mathrm{Max}\;{H\left(\beta \right)|}_{\beta =0}=-25\left(1-c_{R}\right)\left(c_{N}-c_{R}\right)<0\text{.}$ As a result, $\pi_{R}^{C}-\pi_{R}^{B}<0$ always hold.

Similarly, we can proof $\pi_{R}^{N}-\pi_{R}^{B}<0$ always hold.
